# Sustainable Production of Microcrystalline and Nanocrystalline Cellulose from Textile Waste Using HCl and NaOH/Urea Treatment

**DOI:** 10.3390/polym17010048

**Published:** 2024-12-28

**Authors:** Arzum Işıtan, Laura Pasquardini, Massimo Bersani, Cem Gök, Simona Fioravanti, Lorenzo Lunelli, Evren Çağlarer, Ahmet Koluman

**Affiliations:** 1 Department of Mechanical Engineering, Pamukkale University, Denizli 20160, Türkiye; 2 Center for Sensors and Devices, Fondazione Bruno Kessler, 38123 Trento, Italy; bersani@fbk.eu (M.B.); sfioravanti@fbk.eu (S.F.); lunelli@fbk.eu (L.L.); 3 Indivenire srl, 38123 Trento, Italy; l.pasquardini@indiveni.re; 4 Department of Engineering, University of Campania Luigi Vanvitelli, Via Roma 29, 81031 Aversa, Italy; 5 Department of Biomedical Engineering, Izmir Bakırçay University, Izmir 35665, Türkiye; cem.gok@bakircay.edu.tr; 6 Biomedical Technologies Design Application and Research Center, Izmir Bakırçay University, Izmir 35665, Türkiye; 7 Department of Mechatronics Engineering, Kırklareli University, Kırklareli 39100, Türkiye; evren@klu.edu.tr; 8 Department of Biomedical Engineering, Pamukkale University, Denizli 20160, Türkiye; akoluman@pau.edu.tr

**Keywords:** biodegradable polymer, nanotechnology, nanocrystalline cellulose, microcrystalline cellulose, textile waste

## Abstract

Bio-nanomaterials are gaining increasing attention due to their renewable and eco-friendly characteristics. Among these, nanocrystalline cellulose (NCC) stands out as one of the most advanced materials for applications in food, healthcare, composite production, and beyond. In this study, NCC was successfully extracted from cotton-based textile waste using a combination of chemical and mechanical methods. The cellulose fibers were first hydrolyzed using a dilute HCl solution, neutralized, and then dried, resulting in microcrystalline cellulose (MCC) with diameters ranging from 7 to 15 µm and lengths up to 300 µm (as observed via optical microscopy and scanning electron microscopy, SEM). To achieve nanoscale dimensions, NaOH/urea solution with mechanical treatment was applied, resulting in the successful extraction of NCC in the supernatant, particularly under room-temperature conditions. Dynamic light scattering (DLS) analysis confirmed the presence of nanostructures (average sizes ranging from 120 nm to 750 nm), and atomic force microscopy (AFM) analysis verified the nanoscale range (diameters between 2 and 4 nm and lengths from 200 nm to 1 µm). Fourier transform infrared (FTIR) spectroscopy revealed the conversion of cellulose I to cellulose II, confirming the successful transformation into NCC. For the first time, NCC was obtained from undyed cotton textile wastes using NaOH/urea treatment after HCl hydrolysis, eliminating the need for pre-treatment and intermediate steps.

## 1. Introduction

In recent years, there has been a growing demand for materials that are not only environmentally sustainable and human-friendly but also high-performing. This trend has spurred interest in composite materials, which offer both lightness and strength [1,2]. Among these, polymer matrix composites (PMCs) stand out due to their high corrosion resistance, low cost, and lightweight properties. These attributes make them suitable for various applications, including mechanical load-bearing machine components. Polymer-based materials are extensively used across industries, particularly in the medical and automotive sectors. However, these materials face significant sustainability challenges. The increasing carbon footprint, rising energy costs, and difficulties in recycling and disposal present major obstacles. The manufacturing, disposal, and recycling of polymers contribute substantially to global carbon emissions. Moreover, conventional polymers, derived from petroleum, are impacted by the escalating cost of oil.

Polymers, with their long decomposition times, are a major contributor to landfill waste. In the European Union alone, approximately 25.8 million tons of plastic waste are generated annually, with only a small fraction being recycled [3,4]. This situation highlights the urgent need for sustainable, high-performance alternatives to conventional polymers. Composite materials, particularly PMCs, present a promising solution. In recent years, there has been a notable shift towards polymeric materials derived from renewable resources, driven by environmental concerns, rising oil prices, and the limitations of traditional plastic recycling and disposal [5]. Biodegradable biopolymers are increasingly recognized for their potential to address these issues [6,7]. These materials degrade through processes involving UV exposure, microbial action, and enzymatic breakdown, aligning with the principles of a circular economy [5].

Globally, cellulose is the most abundant biopolymer and a primary component of plant cell walls. It is widely utilized in industries such as automotive manufacturing, textiles, coatings, and 3D printing due to its biodegradability, non-toxicity, affordability, and high tensile strength [8]. Common forms of cellulose include microcrystalline cellulose (MCC) and nanocrystalline cellulose (NCC), each possessing unique properties suitable for various industrial applications [9,10]. Given that cotton consists of 95% cellulose, recycling cellulose from post-consumer cotton textile waste offers an innovative approach to fostering circularity in material use [11].

The European Union’s Circular Economy Action Plan (CEAP) and the 2022 EU Strategy for Sustainable and Circular Textiles emphasize waste reduction, resource conservation, and achieving climate neutrality by 2050 [12,13]. Various methods have been proposed for creating composites from textile waste, including the following:

(i) Discarded fabrics as reinforcement;
(ii) Shoddy fibrous materials produced from shredded textile waste;
(iii) Yarn and woven/nonwoven materials generated from textile waste;
(iv) Nano- and microstructures derived from waste textiles [14].

Recycling cellulose fibers and crystals from textile waste with minimal energy input aligns with these goals and offers a sustainable alternative to petroleum-based polymers. NCC can be extracted from cotton-based textile waste using various mechanical, physical, biological, and chemical processes. These include acidic hydrolysis, TEMPO (2,2,6,6-tetramethylpiperidine 1-oxyl) oxidation, ionic liquid methods, deep eutectic solvents, and ammonium persulfate oxidation [15,16,17]. Physical methods involve treatments such as steam or autohydrolysis, hydrothermolysis, aquasolv, uncatalyzed solvolysis, and wet oxidation to break down textile waste into finer particles [15,18,19]. Mechanical methods include shredding and fragmentation [15,20]. Acid hydrolysis is a chemical technique used to obtain microcellulose and nanocellulose from cellulosic fibers [17,21,22,23] using hydrochloric acid (HCl), sulfuric acid (H_2_SO_4_), hydrobromic acid (HBr), nitric acid (HNO_3_), and phosphoric acid (H_3_PO_4_) [15]. Hydrochloric acid (HCl) may be preferred when working with white textiles, and HNO_3_ for dyed textiles [24]. The TEMPO oxidation is used to turn cellulosic fibers into nanocellulose [15]. Derivatizing solvents transform cellulose polymers into soluble intermediates by modifying hydroxyl groups to produce ethers, esters, or acetal derivatives. Because of their low environmental effect, NaOH-based aqueous solutions as cellulose solvents are affordable and might be better suited for industrial-scale applications. Sodium hydroxide (NaOH)/urea aqueous solution has recently emerged as an efficient solvent system, rapidly dissolving cellulose and forming stable solutions [14,25]. Within a limited range of low temperatures and concentrations, MCC can dissolve in aqueous solution at a concentration of 7 to 10% by weight of NaOH in water at −5 to −6 °C. The urea/NaOH aqueous solution works well for dissolving cellulose when it is used at very high concentrations (6–10% NaOH and 8–12% urea) and at low temperatures (−10 °C to −12 °C) [26,27]. Qi et al. [27] showed that by using greater NaOH concentrations and a two-step procedure, cellulose may dissolve in NaOH-based aqueous systems at 0–5 °C.

Cellulose can be obtained from plants and recycled from agricultural, food, wood, and textile wastes. This makes cellulose an antitoxic, environmentally friendly, and sustainable material for the circular economy. MCC is in demand in industrial applications including bulking agents in foods, abrasives in cosmetics, absorbents, binders, anti-cracking agents, bulking agents, emulsion stabilizers, pharmaceuticals, and texturizers [17]. Additionally, the number of studies on nanocellulose has grown exponentially in the past two decades due to its applications in sustainable materials, biomedical devices, and nanotechnology. From 2004 to 2010, fewer than 50 studies per year were published, focusing on the fundamental properties of nanocellulose and its use as a reinforcement material. By 2015, annual publications exceeded 1000, driven by cost-effective production methods and applications in 3D printing, coatings, and packaging. Between 2020 and 2024, annual publications surpassed 3000, fueled by environmental concerns and the adoption of nanocellulose in biodegradable and functional materials [28,29]. One of the major reasons for this is the wide range of applications of NCC. NCC is a highly effective adsorbent for a variety of water-source contaminants, including heavy metals, dyes, oils, and medicines. Its unique characteristics include a high aspect ratio, a wide surface area, and functional groups like hydroxyl and carboxylic groups [30]. Research is being conducted to improve the tribological performance of vegetable oils by producing NCC-added and fully sustainable lubricant compositions [31]. Furthermore, NCC-doped composites significantly enhance the possibility of creating biodegradable films for packaging applications, which might decrease the environmental effect of traditional packaging materials and increase the shelf life of food goods utilizing natural resources [9]. NCC can also find other fields of application in the automotive industry, pulp and paper, medicine and pharmaceuticals such as biomedical implants and drug delivery, barrier/coating and strength additions in nanocomposites in the construction industry, photovoltaics, photonic films, 3D printing, and recyclable electronics [32]. Considering the increasing world population and human needs, it is important to use resources correctly and to transform them after use.

Regarding all production sectors in the world, the textile industry is the fourth category with the highest potential in terms of primary raw material and water use after food, housing, and transportation. At the same time, the textile industry and its products constitute the fifth category in terms of greenhouse gas emissions. Therefore, the utilization of waste generated after use or during production requires urgent action for the textile industry [13]. Despite all this great potential, developing environmentally friendly and cost-effective methods for the extraction and preparation of cellulose derivatives, especially nanocellulose-based materials, remains a significant challenge [29]. Pretreatment methods, chemicals used, and process parameters vary greatly depending on the starting materials and production methods, leaving a single, optimal approach undefined. It is also important to use as minimal chemicals as possible, not to increase energy consumption, and to reduce carbon and water footprints when producing NCC from textile waste.

Considering all these reasons and the previous studies in the literature, in this study, a method with high industrial applicability was investigated by using the least amount of chemicals and energy starting from cotton textile products. To date, no study has been found in which NCC was obtained directly using NaOH/urea after MMC production obtained using HCl solution. In other studies, H_2_SO_4_ or H_2_SO_4_ in combination with HCl was used to obtain NCC, but the amount of H_2_SO_4_ used in these studies was up to 64 wt% [33,34]. In addition, the pre- and intermediate processing steps were numerous. In this study, for the first time, we obtained NCC from undyed cotton textile wastes by using NaOH/urea after HCl hydrolysis by eliminating the pre-treatment and intermediate steps.

MCC was obtained by HCl hydrolysis of two different cotton textile wastes that we use a lot in our lives. MCC was characterized using optical microscopy, SEM, and FTIR. Urea and NaOH solutions were used in combination with mechanical methods to reduce MCC to NCC. During the experiments, process temperatures were varied, and their effects on NCC size were investigated. The resulting NCC samples were analyzed using optical microscopy, SEM, FTIR, AFM, and DLS to determine the optimum intermediate treatment-temperature parameters.

## 2. Materials and Methods

### 2.1. MCC Production

MCC was synthesized through hydrolysis, mixing in a dilute HCl solution, neutralization, and drying processes starting from cotton-based textile waste [35]. Two different starting materials were tested for MCC production. First, experiments were conducted using 100% cotton children’s bodysuits, and MCC was successfully obtained. Subsequently, undyed 100% cotton towels sourced from a textile company in Denizli, Türkiye, were used as the second starting material. Neither material underwent pre-treatment. All chemicals and reagents used in the process were of laboratory grade and unpurified.

In the present study, a commercially available undyed and 100% cotton children’s bodysuit was chosen because it is widely used. In the second stage of the study, 100% cotton towels were used. The textile industry in Denizli province in Türkiye has a history of 4000 years. Known worldwide for the production of cotton towels, bathrobes, and home textiles, Denizli exports to more than 160 countries. There are many production facilities there, and textile recycling is therefore of great importance for Denizli. For this reason, undyed towels produced in Denizli were preferred in this study.

In this study, HCl was chosen for acid hydrolysis in the preparation of MCC. HCl is preferred to obtain MCC and NCC from textile wastes, especially in undyed textiles [22]. Chauhan et al. [36] obtained MCC from old cotton garments and hosiery waste using HCl. In the comparison of the MCC properties obtained in the study with commercial MCC, FTIR, TGA, and XRD results overlapped, and the degree of crystallinity was found to be 81%, which is very close to the commercial MCC of 83%.

**Figure 1:**
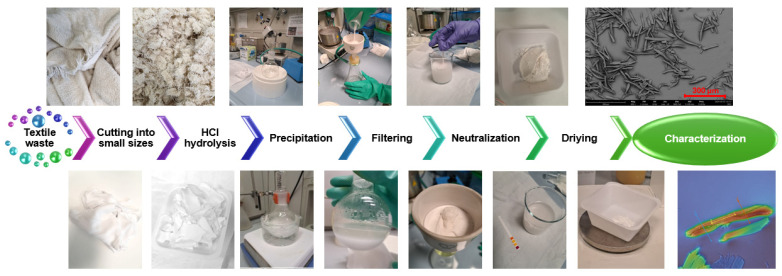
MCC production process.

In the first experiment, 5 g of an undyed children’s bodysuit was cut into small pieces (1 cm × 1 cm) and stirred in 100 mL of 2.5 M HCl final solution (stock solution at 37%) at 100 rpm and 80 °C for 2 h. After dissolution, the solution was left to stand for 16 h at room temperature. The cooled mixture was filtered using filter paper (20–25 µm) and neutralized by washing three times with Milli-Q water ((Milli-Q IQ 7010 purification system from Merk, Milan, Italy) The neutralized MCC, with a pH of approximately 8, was dried at 50 °C for 3 h. Figure 1 summarizes the MCC production steps.

### 2.2. NCC Production

After obtaining MCC, mechanical fragmentation using an ultrasonic probe was first tested as a standalone method to potentially reduce the process steps for NCC production. In this method, 0.3 g of MCC was dissolved in 300 mL of Milli-Q water under magnetic stirring until fully dispersed. The solution was then divided into six 50 mL tubes. Using a Hielscher UP400St ultrasonic mixer (Hielscher Ultrasonics GmbH, Teltow, Germany), energy levels were set to 10,000 Ws, and samples were mixed for 1, 2, 3, and 4 cycles. The resulting solutions were prepared for SEM analysis by placing a drop on a copper tape substrate, which was allowed to dry for 24 h.

For NCC production, except for the ultrasonic treatment, a solution of 7% (*w*/*w*) NaOH and 12% (*w*/*w*) urea was used, similar to the experiment conducted by Abou-Yousef and Kamel [25]. The NaOH/urea system was employed with various raw cellulosic materials, including cotton linter, cellulose pulp, and filter paper, for the production of cellulosic materials [25]. It was observed that the NaOH/urea solution, used for hemicellulose removal from softwood sulfite pulp, directly breaks intermolecular and intramolecular hydrogen bonds in cellulosic fibers, facilitating hemicellulose removal [26].

In the experiments, 1 g of MCC was dissolved in 20 mL of a NaOH:urea solution with a 7:12 (*w*/*w*) ratio (1.4 mg NaOH and 2.4 mg urea) and stirred for 30 min at room temperature. The mixture was frozen at −20 °C for 16 h. After thawing, the contents were stirred at 1000 rpm for 10 min until the MCC was completely dissolved. The regenerated MCC (~180 mL) was obtained by adding ultrapure water (Milli-Q system from Millipore, USA). The solution was centrifuged at 3000 rpm for 10 min to remove urea and NaOH residues. The centrifugation was repeated three times with fresh water until the pH reached 8. The regenerated MCC was suspended in Milli-Q water and ultrasonically treated twice with a 2 mm probe at 75% amplitude for 5 min (Ultrasonic Processor, Cole-Palmer Instruments, Vernon Hills, IL, USA). The resulting NCC was stored at 4 °C until further use.

During initial tests, the solution visibly separated into two phases, showing signs of plasticization and agglomeration in the MCC. Based on this observation, process parameters were adjusted. As mentioned earlier, in order to observe the NaOH/urea effect on MCC, the solution must be kept at low temperatures.

In subsequent experiments, 7% (*w*/*w*) NaOH and 12% (*w*/*w*) urea solutions were kept in an ice bath, with centrifugation performed at 4 °C for NCC extraction. At this stage, an undyed 100% cotton towel obtained from a textile company in Denizli, Türkiye, was used as the starting material. This towel was first subjected to MCC extraction processes: 10 g of towel pieces were cut into small pieces (1 cm × 1 cm) and mixed in a 2.5 M HCl final solution (from a 37% stock solution) for 5 h at 80 °C and 100 rpm.

For further processing, 1 g of MCC was dissolved in 100 mL of NaOH:urea in a percentage ratio of NaOH:urea solution (7% NaOH and 12% urea, 7 mg NaOH, 12 mg urea) and stirred for 30 min in an ice bath. The solution was then divided into three separate tubes, each maintained under different conditions:

(a) Kept in the freezer at −20 °C for 16 h;
(b) Kept at 4 °C for 16 h;
(c) Kept at room temperature (RT) for 16 h.

**Figure 2:**
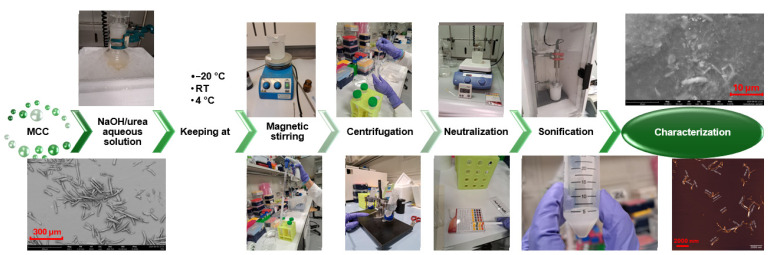
NCC production process.

The different solutions were diluted in Milli-Q water and centrifuged at 3000 rpm for 10 min at 4 °C till a neutral pH was achieved (ALC PK 120R, Zetalab, Italy). After this, mechanical fragmentation was carried out with an ultrasonic tip in an ice bath at 75% amplitude for 5 min (Ultrasonic Processor, Cole-Palmer Instruments, Vernon Hills, IL, USA), repeated twice. The samples used in the study and the processes applied are summarized in Table 1. Figure 2 illustrates the steps involved in the process of obtaining NCC from MCC. 

### 2.3. Characterization of MCC and NCC

#### 2.3.1. Microscopy Analysis

The obtained MCC and NCC samples were characterized using an optical microscope and scanning electron microscopy (SEM). Optical images were obtained using an optical Leica DMLA microscope equipped with a white light in transmission. Images were acquired using a cooled CCD camera (DFC420C, Leica Microsystems, Wetzlar, Germany) and analyzed with the Fiji software version 2.1.0/1.53c (Fiji, ImageJ, Wayne Rasband National Institutes of Health, Bethesda, MD, USA) [37].

SEM imaging was performed with a Phenom XL Desktop instrument equipped with energy dispersive spectroscopy (EDS) to visualize nanocellulose. Samples for SEM were prepared using carbon and copper tape substrates, with some carbon-taped samples gold-coated to a thickness of 30 nm. Imaging and analysis were conducted under varying voltage (5 kV to 15 kV) and vacuum (high to low) settings. Roughness, particle size, and elemental composition analyses were also performed on selected MCC and NCC samples using EDS.

#### 2.3.2. Dynamic Light Scattering (DLS)

Dynamic light scattering (DLS) was employed to measure the size of micro- and nanocellulose particles in the obtained solutions. To minimize interference from excess material, the solutions were diluted 100-fold before analysis. Measurements were conducted using a Zetasizer Nano ZEN3600 with Nano software v3.30 (Malvern Instruments Ltd, Worcestershire, UK), which can measure particle sizes from less than 1 nm to 6 µm. For the analysis, 5 μL of the nanocellulose solution was diluted in 495 μL of Milli-Q. All measurements were performed at room temperature.

#### 2.3.3. Fourier Transform Infrared (FTIR)

FTIR spectroscopy is a technique based on the measurement of the excitation of molecules to vibrational and rotational energy levels using the absorption of IR light (0.78–1000 µm wavelength or 12,800–10 cm^−1^ wave number). Infrared spectra of NCCs were acquired utilizing a Nicolet IN10 microFTIR instrument (Thermo Fisher Scientific, Waltham MA, USA) in transmittance mode. For this purpose, drops of NCC suspensions were deposited on ZnSe optical windows and left to dry in an oven. FTIR analysis was applied to samples MCC1, MCC2, MCC2-RT, MCC2-20, MCC2-4, and MCC1-20.

#### 2.3.4. Atomic Force Microscopy (AFM)

Atomic force microscopy (AFM) was used to obtain surface topography and three-dimensional imaging of the samples. Analyses were performed using an Asylum Re-search Cypher AFM (Oxford Instruments-Asylum Research, Santa Barbara, CA, USA) equipped with an environmental scanner and an Olympus AC240TS probe (Oxford In-struments GmbH Asylum Research, Wiesbaden, Germany)) (nominal force constant 2 N/m, nominal resonance frequency 70 kHz). The instrument was operated in AC acquisi-tion mode, taking data on areas ranging from 400 × 400 nm^2^ to 15 × 15 µm^2^ at a temperature of 25 °C. For sample preparation, a drop of 5 μL of diluted nanocellulose solution was deposited on a freshly cleaved mica substrate and allowed to air-dry at room temperature. These samples were prepared one day prior to analysis. Dilutions of the nanocellulose solution were performed at 10× and 100× ratios in Milli-Q water. The data were analyzed using AFMiJ v 0.1.9a (plugins for opening and analyzing AFM data in ImageJ), an ImageJ (v 1.54k) distribution developed for AFM data analysis [38]. ImageJ is a well-known image-analysis program for scientific applications [37]. Image processing, co-focusing, deconvolution, registration, segmentation, tracking, visualization, and other image analysis methods are made easier with ImageJ. Created by and for the scientific-imaging community, ImageJ is an open-source project on GitHub.

## 3. Results and Discussion

### 3.1. Size and Morphology Analysis by SEM Images and Optical Microscope

The synthesis of MCC involved hydrolysis in dilute HCl, followed by neutralization and drying, using cotton-based textile waste as the raw material. MCC samples were initially examined using an optical microscope and subsequently with SEM for detailed structural analysis.

**Figure 3:**
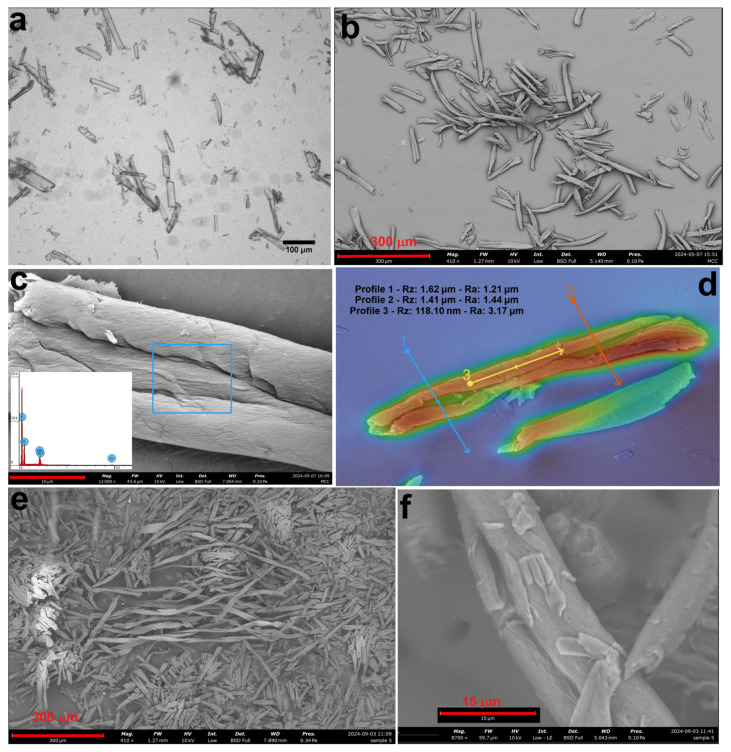
An optical image (**a**), an SEM image (**b**), EDS (**c**) and roughness analyses (**d**) of MCC1, and SEM images of MCC2 at 410× (**e**) and 8700× (**f**) magnifications.

Figure 3 presents optical and SEM micrographs, alongside EDS analysis of the MCC samples. The SEM images confirm that MCC was obtained in the form of short fibers resembling tree bark, with diameters ranging from 7 to 15 µm and lengths up to 300 µm. These results align well with previous studies on MCC structures derived from cotton and textile waste materials [39,40]. Table 2 provides a comparative overview of MCC sizes obtained from various textile products and the corresponding methods reported in the literature.

**Figure 4:**
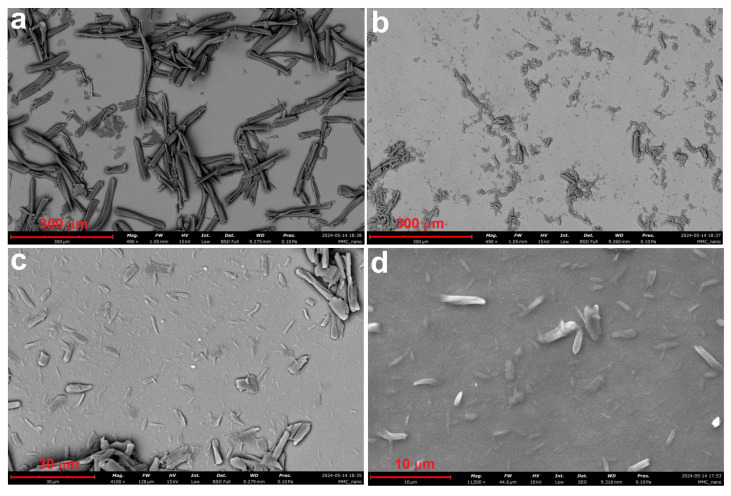
SEM image of sample MCC1-1 subjected to HCl hydrolysis followed by sonication once (**a**), and SEM images at different magnifications of sample MCC1-4 subjected to HCl hydrolysis followed by sonication four times (**b**–**d**).

In samples subjected to mechanical fragmentation (MCC1-1, MCC1-2, MCC1-3, and MCC1-4), SEM analysis revealed progressive fragmentation as the duration and frequency of the sonication process increased. Figure 4 shows the microstructural changes due to sonication HCl hydrolysis: Figure 4a shows the microstructures after one repetition (MCC1-1), and Figure 4b–d show the microstructures after four repetitions (MCC1-4) at different magnifications. The observed size reductions correlate with the increased energy input, confirming that sonication effectively fragments MCC [50].

Traditional nanocellulose extraction methods, which are typically reliant on strong acids like sulfuric acid and energy-intensive mechanical treatments (e.g., high-pressure homogenization), pose significant environmental and economic challenges. In contrast, ultrasound-assisted methods offer a more sustainable alternative by utilizing acoustic cavitation to disrupt lignocellulosic structures. This approach reduces energy and chemical requirements, preserves crystalline integrity, and minimizes agglomeration, yielding higher-quality nanocellulose with better colloidal dispersion. However, ultrasonic methods are not without limitations. The initial concentration of cellulose strongly affects defibrillation efficiency, as higher viscosities impede effective fragmentation. For instance, while a 1% cellulose suspension enhances particle-size reduction, higher concentrations demand increased power input. Additionally, ultrasound generates heat, necessitating cooling systems for thermolabile materials such as lignin-containing nanocellulose. These constraints position ultrasound as a complementary, rather than standalone, method for nanocellulose production [50,51].

Despite its effectiveness, achieving nanoscale dimensions in this study required multiple repetitions of the ultrasonication process, leading to higher energy consumption. This raises concerns about the sustainability of using ultrasound alone for large-scale production. Consequently, it was decided to integrate ultrasonication as a supportive step within chemical processing during NCC extraction.

To address these challenges, an alternative chemical approach was explored. MCC1 was treated with 100 mL of 7% (*w*/*w*) NaOH and 12% (*w*/*w*) urea solution (sample MCC1-20). This process involved solubilization at room temperature, refrigeration at −20 °C for 16 h, centrifugation, and ultrasonication at room temperature.

**Figure 5:**
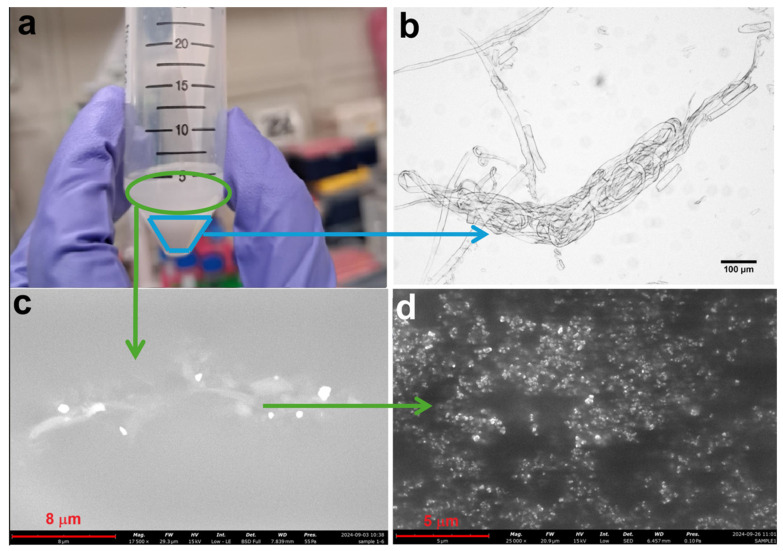
Solution of sample MCC1-20 using NaOH and urea solution (**a**), an optical microscope image taken from the part where precipitation occurred (**b**), and SEM microstructure image from the turbid solution at 17,500× (**c**) and 25,000× (**d**) magnifications.

The results indicated the approach was not entirely effective. The solution visually separated into two phases after treatment (Figure 5a), precipitation was observed at the bottom due to MCC plasticization and agglomeration (light blue line in Figure 5a and optically reported in Figure 5b), while a turbid supernatant remained (green line in Figure 5a), and micro (Figure 5c) and nano (Figure 5d) particles were identified in SEM images. Although the process achieved some nanoscale fragmentation, it was not entirely effective, highlighting the need for further optimization to achieve consistent nanocellulose production.

Aqueous NaOH solutions are widely regarded as the most suitable cellulose solvents for industrial-scale applications due to their low cost and minimal environmental impact. Cellulose fibers can be partially dissolved in NaOH solutions at low temperatures, and the addition of compounds like urea enhances this solubility by facilitating the breaking of hydrogen bonds in cellulosic fibers [25,27]. Urea also interacts with hemicellulose, promoting its disintegration, the breakage of hydrogen bonds in cellulosic fibers, and eventual removal during the NaOH/urea treatment process [26].

To optimize the extraction of NCC while minimizing the formation of precipitates and turbid solutions, the process was performed in an ice bath, and three different holding temperatures were tested: room temperature (RT), −20 °C, and 4 °C. Centrifugation at 4 °C and ultrasonication in an ice bath were also employed to maintain uniformity. For sample MCC1-20, 1 g of MCC1 was dissolved in 20 mL of a NaOH/urea solution prepared at a ratio of 7:12:81. For MCC2, the amount of solvent was increased to 100 mL to increase the supernatant yield and reduce bottom plasticization and turbid solution formation. Revised parameters were applied to specimens MCC2-RT, MCC2-20, and MCC2-4, and their results were compared.

**Figure 6:**
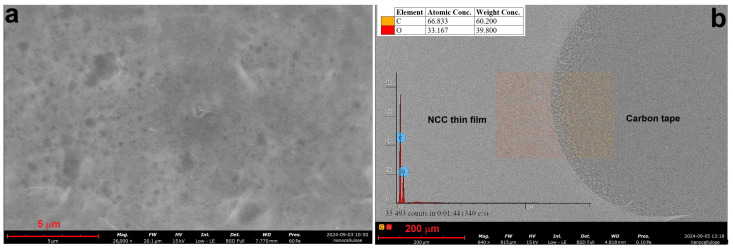
SEM microstructure (**a**) and EDS analysis (**b**) of sample MCC2-RT using NaOH and urea solution.

Figure 6 shows the SEM microstructure of sample MCC2-RT (a) and the corresponding EDS analysis (b). The SEM images reveal NCC structures similar to those reported in the literature [27]. Compared to MCC1-20, MCC2-RT showed improved formation of NCC in the supernatant, with significantly reduced turbidity. Repeated sonication further converted the turbid solution into NCC.

**Figure 7:**
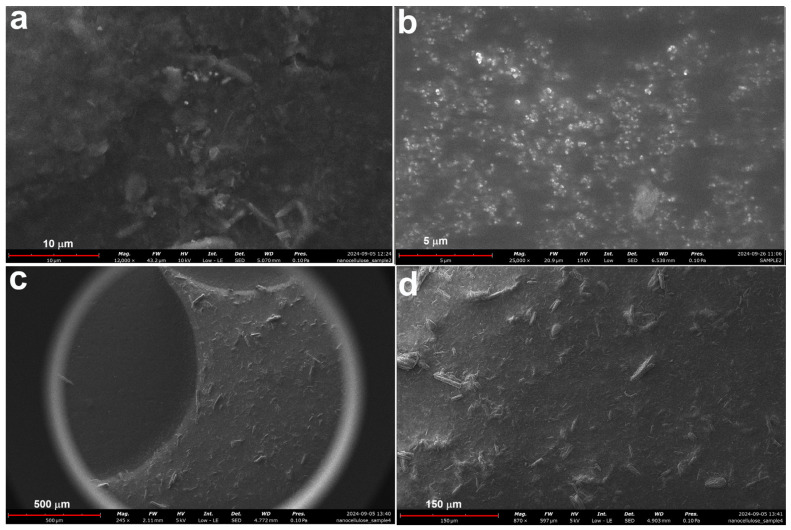
SEM microstructures of sample MCC2-20 (**a**,**b**) and sample MCC2-4 (**c**,**d**) at different magnifications.

Figure 7 illustrates the SEM microstructures of MCC2-20 and MCC2-4. The SEM examination of MCC2-20 showed NCC structures resembling those reported in previous studies [52], although some micro-sized particles were still present. In contrast, a higher proportion of microfibers was detected in MCC2-4. Comparing samples MCC1-20 (Figure 5c) and MCC2-20 (Figure 7a), both processed at −20 °C for 16 h, MCC2-20 exhibited fewer microfibers, indicating an improvement in NCC yield with the revised solvent volume. However, microfibers were still present in both samples, with MCC1-20 showing a higher proportion of microfibers overall. 

A comparison of the sizes and production methods of NCC obtained from textile products and wastes in some studies in the literature with the sizes obtained in this study is presented in Table 3.

The measures taken to increase the amount of supernatant, prevent bottom plasticization, and reduce or eliminate the turbid solution were found to successfully yield nanocrystalline cellulose (NCC) for the MCC2-RT sample. The only difference in processing between MCC2-RT and the MCC2-20 and MCC2-4 samples was that the NaOH/urea soaking and holding steps were performed at room temperature for MCC2-RT rather than at low temperatures (4 °C or −20 °C), as in the other samples.

### 3.2. Size Analysis by Dynamic Light Scattering (DLS)

**Figure 8:**
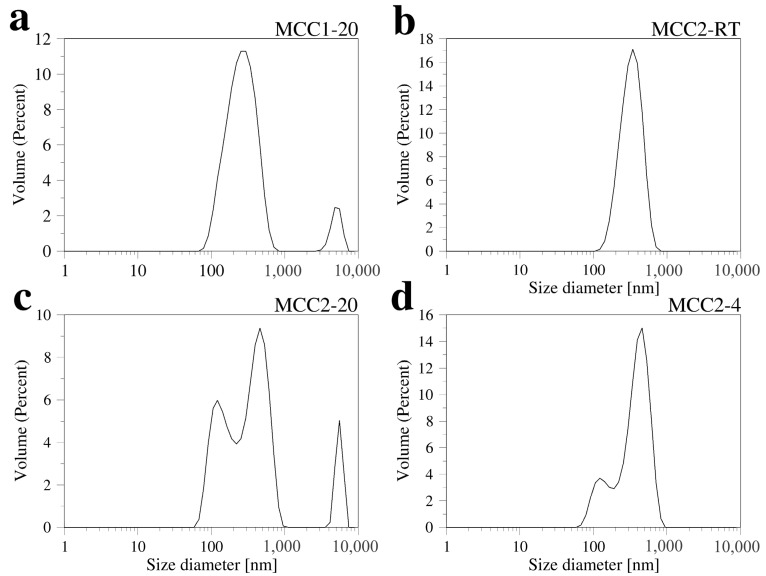
Size distribution of the samples (diluted 100 times from the original colloid): MCC1-20 (**a**), MCC2-RT (**b**), MCC2-20 (**c**), and MCC2-4 (**d**).

Dynamic light scattering (DLS) was used to analyze the particle sizes of nanocellulose in the obtained solutions. Due to the high concentration of material in the initial measurements, the solutions were diluted 100 times, and DLS measurements were performed for samples MCC1-20, MCC2-RT, MCC2-20, and MCC2-4 (Figure 8). These DLS data are interpreted by the analysis software as coming from spherical particles. Its application to long and tiny fibers should be approached with care. The data reported below indicate that there are nanostructures in solution.

Figure 8a shows the DLS results for sample MCC1-20, which indicates a main peak corresponding to a particle size of around 275 nm, with an average size of (z-average diameter) around 231 nm. Also, for MCC2-RT, the DLS results indicate that sub-micrometer-sized particles were achieved.

Figure 8b illustrates the particle-size distribution, with an average particle size of 340 nm. Chattopadhyay and Patel [61] performed size analysis of nanocellulose obtained from waste viscose rayon fibers using the DLS method, and the average size of the particles was found to be 348 nm as a narrow and sharp peak. The particle-size distribution observed for DLS was slightly different from that obtained by AFM (see Section 3.4), which is due to the fact that the DLS analysis software described above is designed for spherical particles, as well as the fact that some NCC particles do not completely dissolve in water, leading to inhomogeneity in the particle-size distribution [60].

Figure 8c displays the DLS results for sample MCC2-20, which shows two size-distribution peaks in the sub-micrometer range. Peak 1 records a particle size of 141 nm, and Peak 2 records 440 nm. The average size (z-average diameter) is 561 nm, possibly due to some contamination (represented by the third peak in Figure 8c). Finally, Figure 8d presents the DLS results for sample MCC2-4, which also shows two size-distribution peaks: Peak 1 records 426 nm, and Peak 2 records 133 nm, with an average size of 504 nm.

Pandi et al. [52] performed particle-size distribution analysis of NCC obtained from various acid concentrations and ultrasound treatment using the DLS method [45]. The analysis showed that the majority of the particles were in the size range of 100 to 800 nm, with smaller particles in the size range of 20 to 100 nm. Lim et al. [62] performed size analysis of nanocellulose obtained from oil-palm empty fruit bunches using the DLS method (with SEM support) and obtained two size-distribution intensities represented by two peaks [56]. An average size of 561.0 nm was obtained. However, when NCC was examined by TEM and AFM, it was determined that the structure was rod-like with lengths ranging from 100 nm to 3000 nm and widths ranging from 3 nm to 30 nm.

### 3.3. Fourier Transformed Infrared (FTIR) Analysis

**Figure 9:**
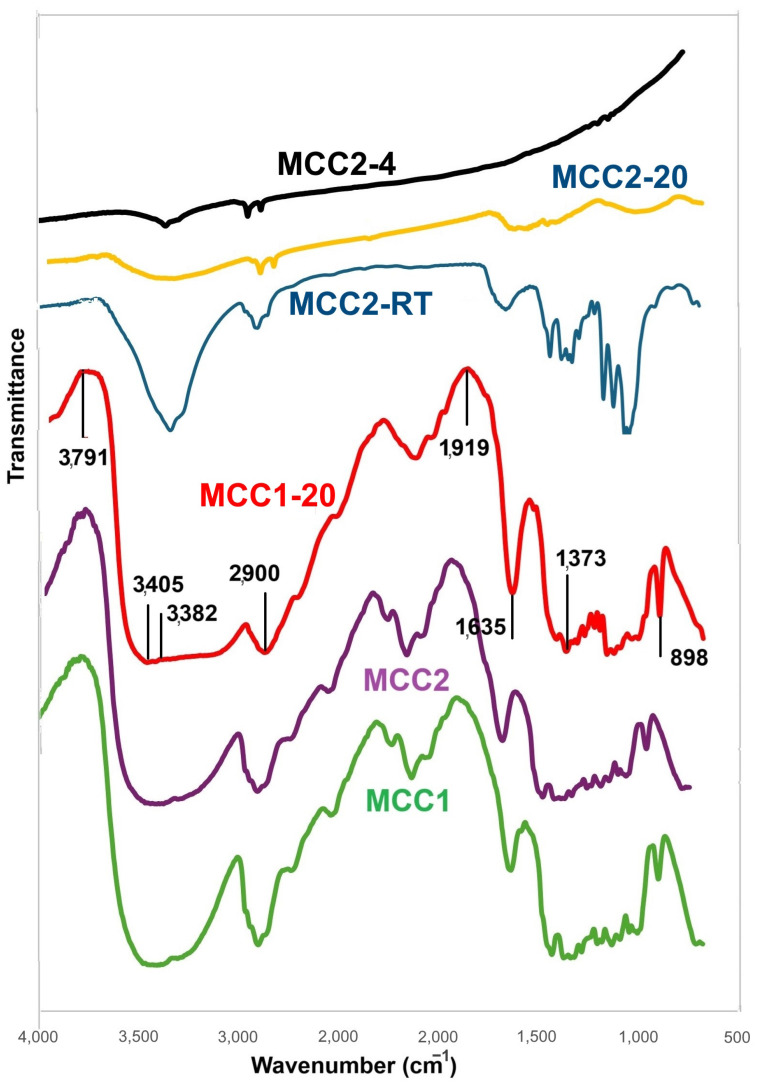
FTIR spectra of all samples.

Samples MCC1, MCC2, MCC1-20, MCC2-RT, MCC2-20, and MCC2-20 were characterized using FTIR analysis. Figure 9 represents the FTIR spectra of the samples. MCC1, MCC2, and MCC1-20 show the same spectra. Spectra at 4000–3004 cm^−1^, 2900 cm^−1^, 1407 cm^−1^, 1373 cm^−1^, and 898 cm^−1^ indicate crystal and amorphous regions. There are structure changes of cellulose I to cellulose II with NaOH 7%, which is shown by stretching OH at 3382 cm^−1^ and stretching C=O at 1635 cm^−1^. The characteristics of the glycosidic group of C-O, C-O-C, and C-OH bonds with concentration variations are seen at 1164 cm^−1^. The formation of alkali-cellulose is reinforced by stretching of the C-O and C-C groups at 1060 cm^−1^. There is a sharp peak at 898 cm^−1^, which is typical for β-glycosidic linkages of cellulose I. There are multiple kinds of cellulose found in nature, including cellulose I, II, III, and IV [63]. Cellulose II is chemically regenerated in laboratories, whereas cellulose I is thought to be a native cellulose type [41]. Samples showed peak shifts at 995 cm^−1^. These peaks correspond to the cellulose spectrum [64,65]. The results for samples MCC2-20 and MCC2-4 were not insightful due to the low amount of material.

**Figure 10:**
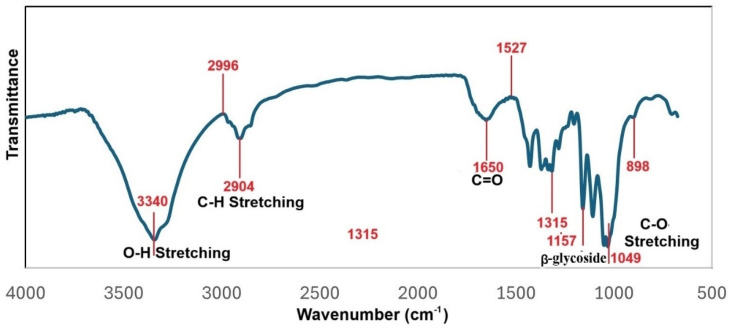
FTIR spectra of sample MCC2-RT.

The FTIR analysis obtained for the MCC2-RT sample detailed in Figure 10 differed from all other samples. Peaks at 3340 and 2904 cm^−1^ were observed in the MCC2-RT, MCC2-20, and MCC2-4 samples due to stretching and symmetrical stretching of -OH and C-H, respectively. In Figure 10, the peak at 898 cm^−1^ represents the C-H deformation in cellulose. The band at 1157 cm^−1^ is characteristic of glycosidic groups and is attributed to C-O and C-O-C stretching and C-OH bending vibrations in the arabinoxylan structure. In addition, a sharp absorption peak at 1106 cm^−1^ in cellulose II is also indicative of high cellulosic content [66,67,68]. In particular, NCC showed an intense peak at 1650 cm^−1^, which is attributed to the C=O stretching of the carbonyl of carboxylic acid groups corresponding to the presence of cellulose [69].

### 3.4. Atomic Force Microscopy (AFM) Analysis

**Figure 11:**
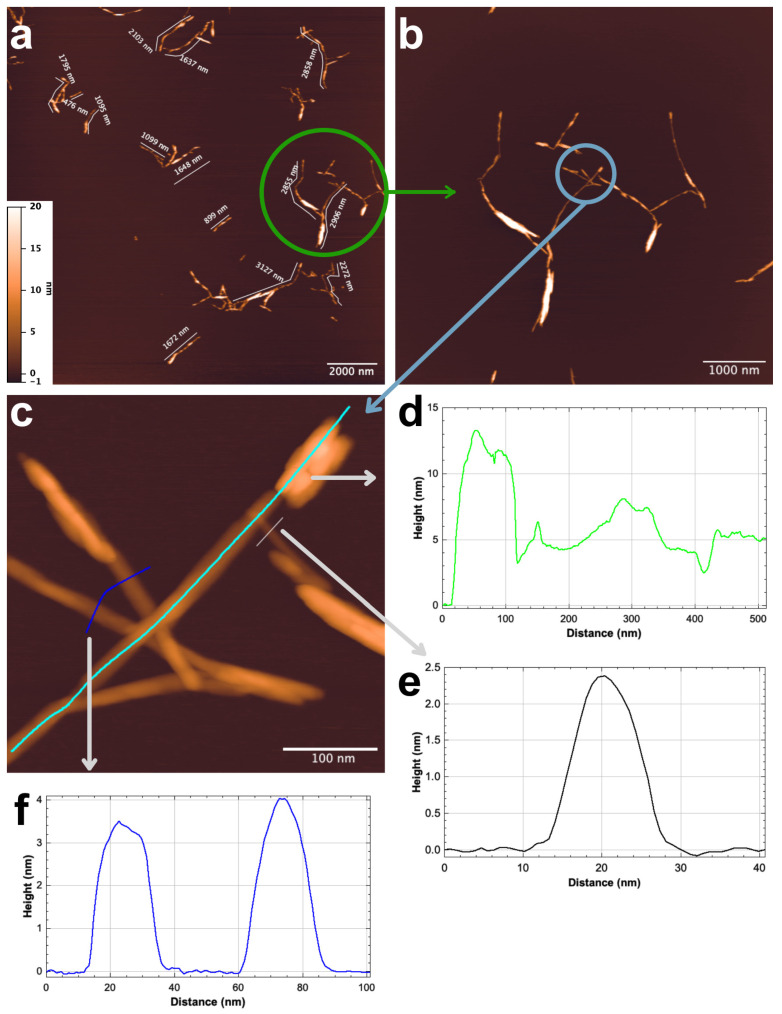
Sample MCC2-RT’s AFM visual images (**a**,**b**), NCC fiber measurement examples (**c**), and fiber measurement results (**d**–**f**).

Based on the SEM, DLS, and FTIR results, it was concluded that MCC2-RT had a high amount of NCC. Therefore, AFM analysis was performed on MCC2-RT to assess the morphological, topological, and size features of the prepared NCC. Measurements were taken on at least 100 individual nanofibers from the AFM images. The AFM analysis confirmed the production of NCC with diameters ranging from 2 to 4 nm and lengths between 200 nm and 1 µm, characterized by a short fiber structure. Figure 11 displays representative images and size distributions of NCC characterized by a short fiber structure.

A crucial NCC characteristic that affects the performance of suspensions, functional materials, and NCC-reinforced polymers is the aspect ratio, or length divided by diameter (L/d). It was previously shown that the higher aspect ratios of NCC contribute to a better reinforcing effect due to the enlargement of the interfacial area, a strong reinforcing effect that can be very important in improving the physical properties of composite materials [34,70]. The findings (diameters ranging from 2 to 4 nm and lengths between 200 nm and 1 µm) were compared to previous studies. In the study by Mohamed et al. [61], NCC was isolated from waste-cotton cloths collected from a landfill. The NCC was isolated using H_2_SO_4_ hydrolysis of cellulose. It was found that the particle size of isolated NCC varied from 37.84 to 342 nm, with an average particle size of 109.1 nm. Similarly, Wang et al. [34] isolated NCC from waste-cotton cloth using alkali and bleaching treatments, followed by mixed-acid-solution hydrolysis. The sizes of the NCC ranged from 28 to 470 nm in length and 3 to 35 nm in diameter. In addition, as can be seen from Table 3, the aspect ratio obtained in this study is higher than in the comparative literature. In summary, the NCC production proposed in this study is not only easy to implement but also has very high potential for use as composite reinforcement.

## 4. Conclusions

This study indicates that MCC from cotton textile wastes obtained by HCl hydrolysis can be effectively extracted as nanocrystalline cellulose (NCC) by NaOH/urea chemical treatment followed by ultrasound-assisted degradation. Many pre- and intermediate treatments applied in the literature were eliminated by varying the temperatures of soaking, centrifugation, and sonication processes for NCC extraction. The effects of these temperatures on NCC size were revealed by SEM, DLS, and FTIR analysis. Of these parameters, the one with the largest positive effect was found to be room temperature, which is the holding temperature after the soaking reaction in NaOH/urea solution, resulting in lower energy consumption compared to other temperature parameters.

The SEM images revealed that MCC was obtained as short fibers with diameters ranging from 7 to 15 µm and lengths up to 300 µm. Characterization results demonstrated the effectiveness of the applied process with NaOH/urea, with AFM analysis revealing NCC structures with diameters ranging from 2 to 4 nm and lengths between 200 nm and 1 µm. FTIR analysis further supported these findings, showing the conversion of cellulose I to cellulose II, a key structural transformation in nanocellulose. These findings highlight the potential of cotton textile waste as a sustainable source for NCC production using the least amount of chemicals and energy, contributing to waste reduction but also in line with the growing interest in bio-based materials for various industrial applications. Further studies may investigate the scalability of these methods and their potential for large-scale production of biopolymeric materials from renewable resources.

## Figures and Tables

**Table 1:** The starting materials used in the study and the process parameters applied.

Starting Material	Sample	NaOH and Urea Solubilization in an Ice Bath	Kept Time and Temperature	Centrifugation Temperature	Sonication in an Ice Bath
Children’s clothing	MCC1	---	---	---	---
Undyed towel	MCC2	---	---	---	---
MCC1	MCC1-20	No	−20 °C for 16 h	RT	---
	MCC1-1	No	−20 °C for 16 h	RT	No
	MCC1-2	No	−20 °C for 16 h	RT	No
	MCC1-3	No	−20 °C for 16 h	RT	No
	MCC1-4	No	−20 °C for 16 h	RT	No
MCC2	MCC2-RT	Yes	RT for 16 h	4 °C	Yes
	MCC2-20	Yes	−20 °C for 16 h	4 °C	Yes
	MCC2-4	Yes	4 °C for 16 h	4 °C	Yes

**Table 2:** Some methods and dimensions for obtaining MCC from textile wastes.

Raw Materials	Length (µm)	Width/Height/ Diameter (µm)	Methods	References
100% polyester yarn with fresh raw cotton fibers	45–65	20–30	H_2_SO_4_ hydrolysis	[41]
Cotton sliver	5	--	H_2_SO_4_ hydrolysis	[42]
Cotton sliver	1–1900	0.15–0.020	H_2_SO_4_ hydrolysis	[43]
Waste cotton:polyester ~19:1 clothes	0.5–1	--	H_2_SO_4_ hydrolysis	[17]
Yarn waste	27	--	H_2_SO_4_ hydrolysis	[44]
Cotton waste	--	37.8	Alkaline treatment and H_2_SO_4_ hydrolysis	[45]
Cotton/polyester fabric blends	45–65	20–30	Chemical pretreatment and H_2_SO_4_ hydrolysis	[41]
Waste-cotton dyed cotton shirt	0.87–111	--	Phosphotungstic acid hydrolysis	[46]
Jute fabric	14.28–181.31	2.197–63.73	Alkaline treatment with NaOH	[47]
Cotton garment fabric waste	--	32	HCl hydrolysis	[48]
Yarn waste	25	--	HCl hydrolysis	[44]
Short staple cotton variety: Bengal desi	45–53	--	HCI hydrolysis	[33]
Waste-cotton fabrics	--	20–50	HCI hydrolysis	[49]
Cotton children’s clothing	50–300	7–15	HCl hydrolysis	Present study
Cotton towel	50–400	12–17	HCl hydrolysis	Present study

**Table 3:** Dimensions of NCC obtained from textile products and wastes and the methods of their production.

Raw Materials	Length (nm)	Height/Diameter (nm)	Methods	References
Commercial cotton balls	150 ± 50	14 ± 5	Alkali extraction, H_2_SO_4_ hydrolysis, centrifugation, neutralization, ultrasonication	[53]
Cotton-stalk bleached pulps	450	25	Treated with synthetic white liquor, bleached cotton pulp using hydrogen peroxide, H_2_SO_4_ hydrolysis, sonification, centrifugation	[54]
Short staple cotton variety: Bengal desi	287.24 ± 79.75	29.69 ± 5.07	MMC by HCl hydrolysis; H_2_SO_4_ hydrolysis, centrifugation, neutralization, freeze-dried	[33]
	120.27 ± 36.25	40.74 ± 7.59	Enzymatic hydrolysis	
Cotton fabrics	76–159	14.2–15	NaOH solution treatments, neutralization, H_2_SO_4_ hydrolysis, centrifugation, neutralization, high-pressure homogenization, ultrasonication	[55]
Cotton linters	177	12	H_2_SO_4_ hydrolysis, neutralization, centrifugation	[56]
Degreasing cotton	17–230	2–25	Boiled with NaOH, neutralization, mixed H_2_SO_4_ and HCI hydrolysis, neutralization, sonification, centrifugation	[34]
Waste-cotton cloth	28–470	3–35		
Textile waste from factory	111.76 ± 38.73	11.18 ± 2.33	H_2_SO_4_ hydrolysis	[57]
	97.25 ± 25.18	5.69 ± 2.08	Three-step oxidization	
Post-consumer cotton fabrics	218 ± 112	12 ± 7	Citric-acid esterification, sonification, neutralized by NaOH, fibrillated using high-pressure microfluidization	[58]
MCC from waste-cotton fabrics	5–100	10–65	MCC obtained by boiling NaOH, boiling with NaClO, and nitric acid and HCl hydrolysis; H_2_SO_4_ hydrolysis, neutralization, sonification, centrifugation	[59]
Waste cloths from landfill	10–30	2–6	Alkali pulping with NaOH, bleaching, and H_2_SO_4_ hydrolysis, centrifugation, homogenization, ultrasonication	[60]
MCC from cotton towel by HCl hydrolysis	200–1000	2–4	NaOH/urea solvent system and ultrasonication	Present study

## Data Availability

Data are contained within the article.
